# Montelukast, an available and safe anti-asthmatic drug, prevents maladaptive remodelling and maintains cardiac functionality following myocardial infarction

**DOI:** 10.1038/s41598-024-53936-x

**Published:** 2024-02-09

**Authors:** Majeda Muluhie, Laura Castiglioni, Joanna Rzemieniec, Benedetta Mercuriali, Paolo Gelosa, Luigi Sironi

**Affiliations:** Department of Pharmaceutical Sciences, University of Milan, Via G. Balzaretti 9, 20133 Milan, Italy

**Keywords:** Animal disease models, Heart failure

## Abstract

Preclinical and clinical data indicate that the 5-lipoxygenase pathway becomes activated in cardiovascular diseases suggesting an important role of CysLTs in atherosclerosis and in its ischemic complications. This study aims to investigate the effects of montelukast, a CysLTR-1 antagonist, in a mouse model of myocardial infarction (MI). C57BL/6N female mice were subjected to coronary artery ligation and received montelukast (10 mg/kg/day, intraperitoneal) or vehicle. Montelukast exerted beneficial effects in the infarcted area, decreasing mRNA expression of inflammatory genes, such Il1β and Ccl2 (p < 0.05), at 48 h after MI, and reducing infarct size and preventing ischemic wall thinning (p < 0.05) at 4 weeks. Furthermore, montelukast counteracted maladaptive remodelling of whole heart. Indeed, montelukast reduced LV mass (p < 0.05) and remote wall thickening (p < 0.05), and improved cardiac pumping function, as evidenced by increased global ejection fraction (p < 0.01), and regional contractility in infarcted (p < 0.05) and in remote non-infarcted (p < 0.05) myocardium. Finally, montelukast prevented cardiomyocytes hypertrophy (p < 0.05) in remote myocardium, reducing the phosphorylation of GSK3β, a regulator of hypertrophic pathway (p < 0.05). Our data strongly demonstrate the ability of montelukast to contrast the MI-induced maladaptive conditions, thus sustaining cardiac contractility. The results provide evidences for montelukast “repurposing” in cardiovascular diseases and in particular in myocardial infarction.

## Introduction

Drug repurposing is a strategy for identifying—for already approved drugs—new uses that are outside the scope of the original medical indication. This strategy offers several advantages over developing a completely new drug for a given indication reducing time and cost for the preclinical studies and safety profiling, and cutting down time for approval^1^. Moreover, the risk of failure in reposition is low because the drug has already been found to be sufficiently safe in both preclinical and clinical studies. In clinical practice there are many examples where repositioning has happened successfully, such as thalidomide and sildenafil, and from 2012 to 2017, almost 170 repurposed drugs entered the drug development pipeline^2^ indicating how this approach is very promising.

In this respect, new opportunities seem to derive from the repurposing of antagonists of cysteinyl-leukotrienes (CysLTs) receptor 1, such as montelukast, for treatment of cardiovascular diseases (CVDs). Indeed, CysLT involvement has been hypothesized in acute myocardial infarction (MI), ischemic stroke, atherosclerosis, aortic aneurysms, and intimal hyperplasia^3–5^. Several preclinical observations suggest that the anti-inflammatory properties of this class of drugs go beyond pulmonary disease, exerting beneficial effects in a number of cardio- and cerebrovascular pathological processes. In particular, montelukast reduced ox-LDL-induced adhesion of monocytes to endothelial cells, suppressing VCAM-1 and E-selectin expression^6^, delayed the progression of atherosclerosis and intimal hyperplasia^7^ by inhibiting the expression of monocyte chemoattractant protein 1 (MCP-1)^8^ and matrix metalloproteinases (MMPs)^9^, and prevented aortic dilatation, rupture and aneurism development^10^. Relevantly, CysLTR-1 expression is increased in response to the released pro-inflammatory cytokines from coronary atherosclerotic lesions^11^. At cerebrovascular level, montelukast protected against disruption of brain endothelial junction proteins, suppressing the expression and biosynthesis of MMPs and cytokines^12^, and exerted protective effects after cerebral ischemia^3,13,14^.

In humans, a study on a cohort of patients provided a first indication of CysLTR-1 antagonists for the secondary prevention of CVDs, including ischemic stroke^15^. Recently, an observational retrospective study reported a significant relationship between the use of montelukast and the reduction of a major ischemic CV event, such as MI or ischemic stroke, in asthmatic patients^16^. In this population, an interventional study demonstrated that montelukast can reduce the levels of CVD-associated inflammatory biomarkers, such as C-reactive protein, and lipid levels^17^.

However, the data available on a possible effect of CysLTs receptor antagonists at cardiac level are limited. Montelukast reduced myocardial hypoxic area^18^, improved cardiac function after cryo-induced myocardial damage^19^ and attenuated myocardial injury induced by lipopolysaccharide (LPS)^20^ and arsenic trioxide^21^, possibly by its anti-inflammatory and antioxidant effects. Moreover, in mouse transverse aortic constriction (TAC) model, montelukast improved cardiac functional and morphological alterations^22^.

Here, we investigated, taking advantage by the use of cardiac Magnetic Resonance Imaging, the gold standard technique for in vivo cardiac imaging, the effects on cardiac functional parameters of the administration of montelukast in a rodent model of MI induced by the permanent ligation of left anterior descending coronary artery. The goal of the study is to understand if, the anti-inflammatory and anti oxidizing effects of montelukast—already described in literature—translate into an effective improvement in cardiac function that is the most important aspect to support its investigation in clinical studies.

## Results

### Cytokine and apoptotic gene expression

At 48 h after LAD ligation, the MI group exhibited considerable high amounts of mRNA for Il1β, TNF$\mathrm{\alpha }$ and Ccl2 in the left ventricle infarcted wall compared to SHAM group (p < 0.05) (Table 1). Montelukast treatment completely prevented the enhanced mRNA expression of Il1β and Ccl2 (p < 0.05), and, although it has not reached the statistically significance, also reduced the mRNA expression of TNF$\mathrm{\alpha }$ (Table 1).

**Table 1:** Gene expression 48 h after surgery.

Gene	SHAM	MI	MI + MTK
Il1β	1.00 ± 0.36*	3.37 ± 0.95	0.83 ± 0.13*
Ccl2	1.00 ± 0.52*	4.85 ± 1.19	1.61 ± 0.22*
TNFα	1.00 ± 0.10	2.10 ± 0.80	1.18 ± 0.13
Bcl-2	1.00 ± 0.07	0.60 ± 0.05	1.50 ± 0.41*
BAX	1.00 ± 0.12*	1.45 ± 0.14	0.83 ± 0.07**
BAX/Bcl-2	1.00 ± 0.04**	2.45 ± 0.48	0.80 ± 0.22**
NPPA	1.00 ± 0.57*	3.55 ± 0.62	1.51 ± 0.31*
NPPB	1.00 ± 0.26*	3.81 ± 0.92	0.78 ± 0.24**
MHY7	1.00 ± 0.29	1.63 ± 1.07	0.56 ± 0.18

Gene levels are expressed as 2^−ΔΔCt^ values. Data are expressed as mean ± SEM. One-way ANOVA analysis followed by Dunnett's Multiple Comparison Test, * p < 0.05 versus MI.

Moreover, the exposure to LAD ligation induced a significant increase (p < 0.05) in the pro-apoptotic BAX gene expression and a decrease in the anti-apoptotic Bcl-2 gene expression, in the LV infarcted wall compared to SHAM group. Montelukast treatment was able to protect tissue from apoptotic process, significantly preventing the increase and decrease of BAX (p < 0.01) and Bcl-2 (p < 0.05), respectively. The BAX/Bcl-2 ratio resulted to be significantly (p < 0.01) increased in MI group but decreased upon montelukast treatment (p < 0.01) (Table 1).

### Cardiac remodelling and function

The cMRI images were analysed in order to determine global left ventricular (LV) parameters as end-diastolic volume (EDV), end-systolic volume (ESV), ejection fraction (EF%) and LV mass. Furthermore, wall thickness (WTh) and regional fractional area change (RFAC), an index of regional contractility, were measured in order to evaluate the regional remodelling of infarct, border and remote non-infarcted zone.

Compared to SHAM group, 24 h after LAD ligation, the infarcted groups (MI and MI + MTK) exhibited severe LV dilatation showing an increase in EDV (p < 0.05) and ESV (p < 0.001), and a concomitantly significant reduction of global EF% (p < 0.001) and regional contractility of remote non-infarcted region (p < 0.001). At this time point, no difference was observed in LV mass and posterior wall thickness (Table 1S).

In acute phase at 48 h after myocardial infarction we observed a significant increase of hypertrophic genes as ANP and BNP in the non-infarcted LV wall compared to SHAM (p < 0.05 for both, Table 1). These alterations were completely prevented by montelukast treatment (p < 0.05 for ANP and p < 0.01 for BNP, Table 1). Although not achieving statistical significance, similar data was obtained for mRNA MHC.

**Figure 1:**
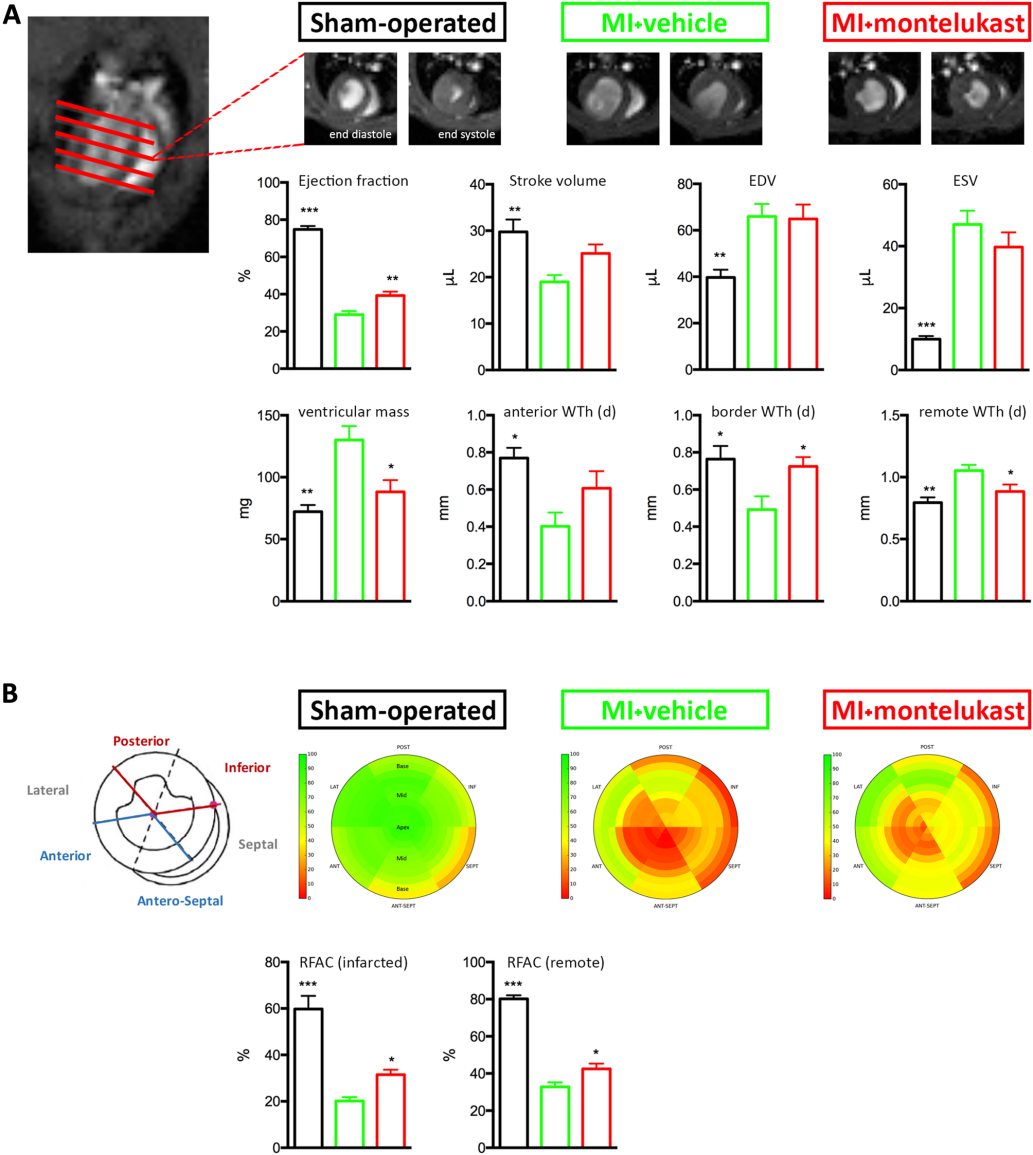
Montelukast improves post-MI cardiac remodelling, attenuating the development of left ventricular hypertrophy and preserving regional contraction. (**A**) On the left, a representative long-axis cardiac MRI image of a SHAM-operated mice. The red lines represent the position of the slices acquired. On the right the short-axis cardiac MRI images (end-diastolic and end-systolic mid papillary frames) taken from a SHAM-operated, MI or MI + MTK mice. Quantitative assessment of LV remodelling using established measures of ejection fraction (%), stroke volume, end-diastolic and systolic volume, LV mass, and wall thickness (in the anterior, border and remote zone). (**B**) Bull’s eye representations of mean regional fractional area change (RFAC) in SHAM, MI and MI + MTK groups at 4 weeks after MI (n = 5–6/group). Slices from LV apex to the base are shown from the inner to the outer circle, whereas red and green tones indicate lower and higher RFAC values (the color bar on the right shows the corresponding scale). Lat: lateral; Post: posterior; Inf: inferior; Sept: septal; Ant-Sept: Antero-septal; Ant: anterior. Quantitative assessment of RFAC (%). Data are expressed as mean ± SEM. One-way ANOVA analysis followed by Dunnett's Multiple Comparison Test, * p < 0.05; ** p < 0.01 and *** p < 0.001 versus MI.

At 4 weeks after LAD ligation, we observed a significant enlargement of LV cavity in terms of EDV (p < 0.01) and ESV (p < 0.001) and a significant reduction of global EF% (p < 0.001), stroke volume (p < 0.01) (Fig. 1A) and RFAC% in the ischemic and remote zone (p < 0.001) (Fig. 1B) in MI group compared to SHAM group. Concomitantly, we observed a significant increase of LV mass (p < 0.01) and diastolic posterior wall thickness (p < 0.01), while a decrease of diastolic wall thickness in the ischemic and border zone (p < 0.05) (Fig. 1A).

Montelukast significantly increased the global EF% compared to vehicle treatment (p < 0.01), although the improvement in stroke volume, EDV and ESV were not statistically significant (Fig. 1A). Relevantly, the analysis of regional LV function showed a significant protective effect in terms of contraction property exerted by montelukast not only in the remote non-ischemic wall, but also in the anterior ischemic wall (Fig. 1B). These functional effects were associated with a finest post-MI remodelling. In fact, montelukast significantly decreased LV mass (p < 0.05) and the diastolic wall thickness in the remote zone (p < 0.05) compared to vehicle treatment, while preserved the thinning of the wall in the border zone (p < 0.05) and in the anterior ischemic zone, although for the latter it has not reached the statistically significance (Fig. 1A).

### Histological analysis

**Figure 2:**
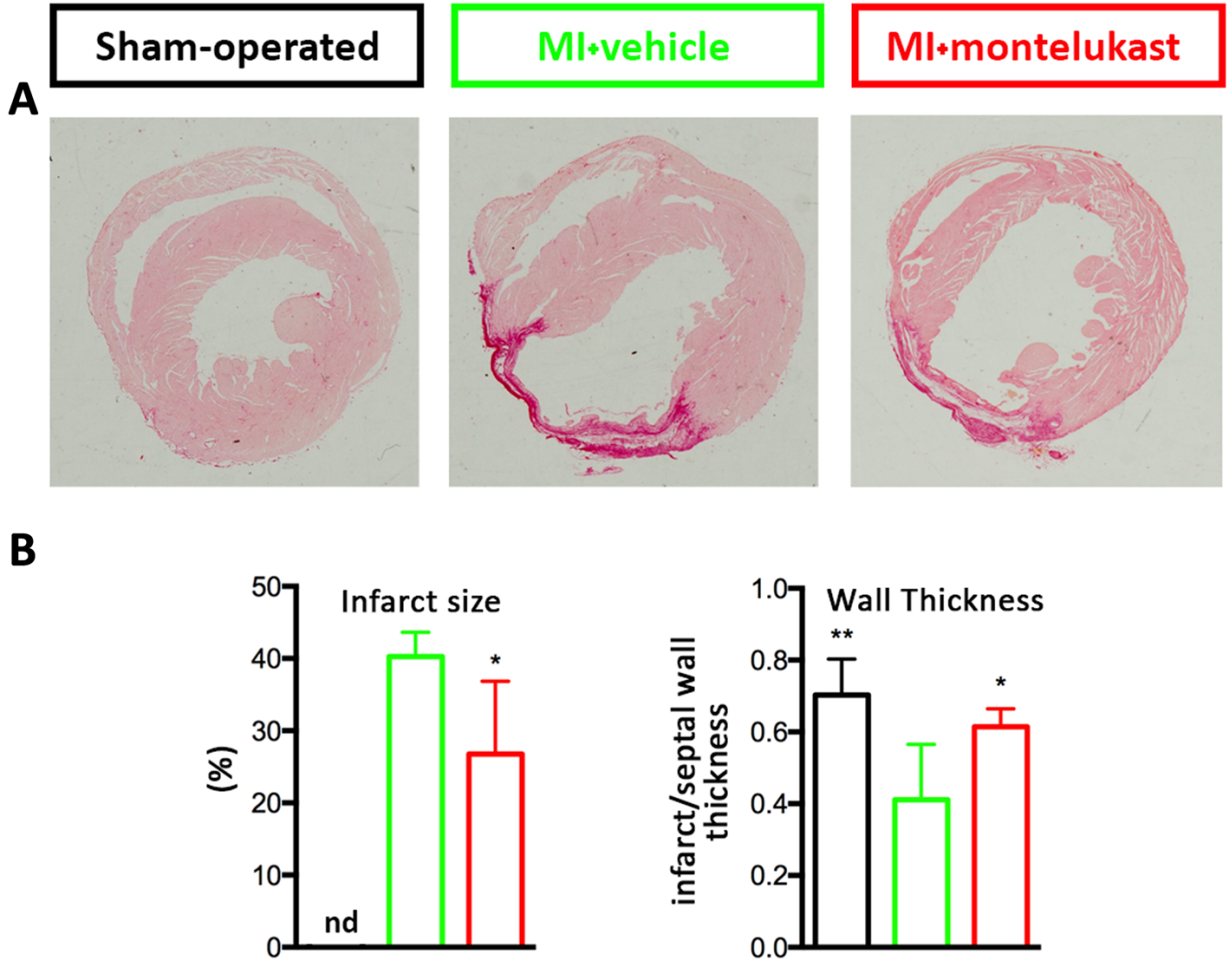
Montelukast reduces infarct size and wall thinning. (**A**) Representative images of Sirius Red staining of cardiac axial section from SHAM, MI and MI + MTK mice showing left and right ventricular chamber at week 4 post-surgery. (**B**) The bar graphs are related to quantitative assessment of infarct size and wall thickness of infarct scar at papillary muscle level (n = 3–5/group). Infarct size is not detected (nd) in SHAM group. Data are expressed as mean ± SEM. For infarct size unpaired t Test and for wall thickness One-way ANOVA analysis followed by Dunnett's Multiple Comparison Test were performed, * p < 0.05; ** p < 0.01 and *** p < 0.001 versus MI.

At 4 weeks after LAD ligation, Sirius Red stain showed that the infarct size was significantly reduced in MI + MTK compared to MI group (p < 0.05; Fig. 2. Moreover, the infarct wall was thicker (p < 0.05; Fig. 2), confirming the data obtained by cMRI analysis, in MI + MTK compared to MI group.

**Figure 3:**
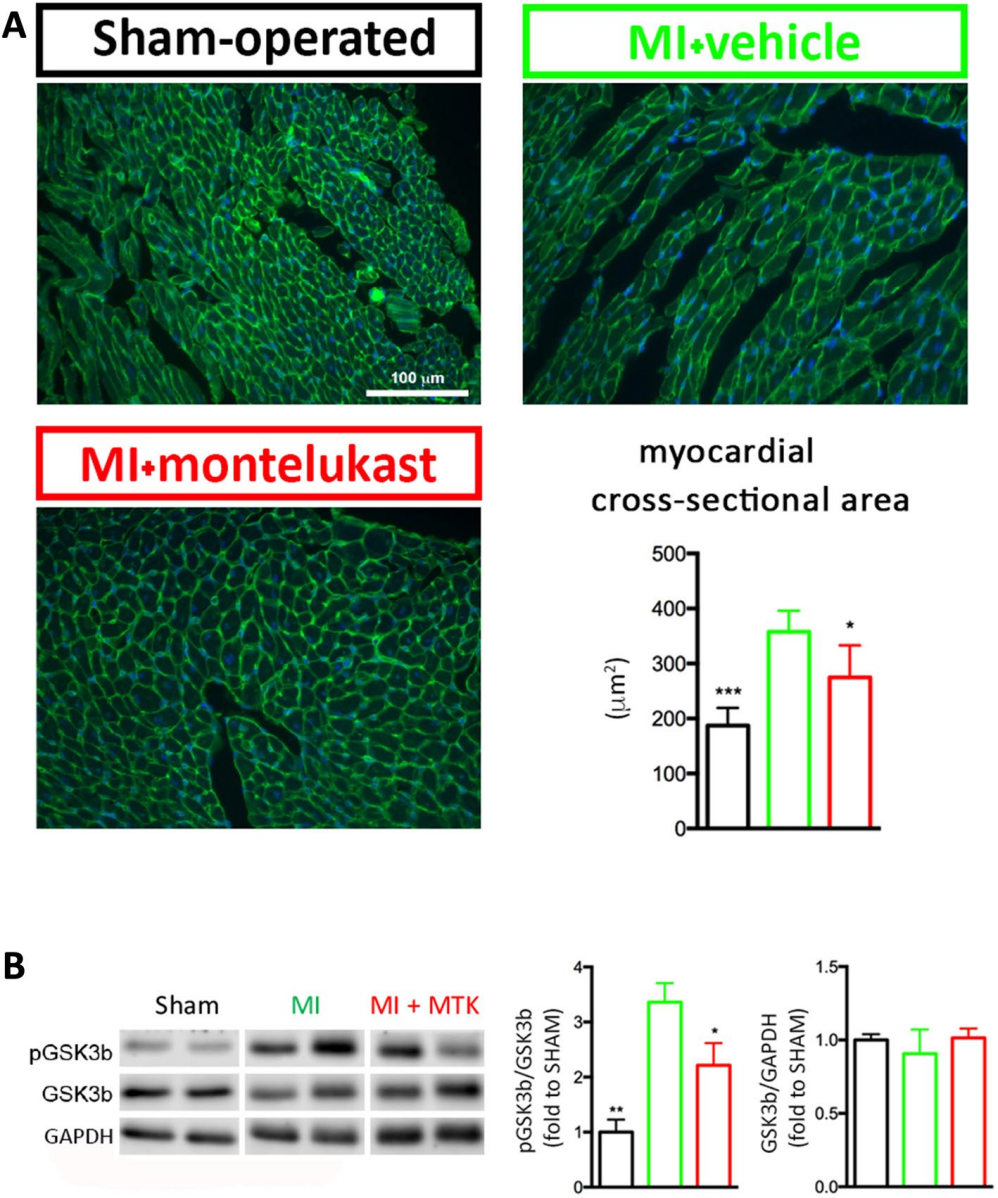
Montelukast prevents hypertrophy of cardiomyocytes. (**A**) Representative images of wheat germ agglutinin staining in the remote myocardium from SHAM, MI and MI + MTK mice at week 4 post-surgery. Scale bar 100 $\upmu$m. The bar graph is related to the cross-sectional area (CSA), which was significantly counteracted by montelukast (n = 3–5/group). (**B**) Western blot analysis of GSK3β of cardiac sample from remote zone at week 4 post-surgery (n = 3–6/group). The blots were cropped from different parts of the same gel as delineated with dividing white space. The original images of full-length blots cannot be provided because the blots were cut prior to hybridisation with antibodies. Gels and blots with molecular size marker and membrane edges visible are presented in supplementary information and the regions of the original blots used in main figures were denoted using red boxes (Fig. 1S–5S). Data are expressed as mean ± SEM. One-way ANOVA analysis followed by Dunnett's Multiple Comparison Test, * p < 0.05; ** p < 0.01 and *** p < 0.001 versus MI.

Histological analysis performed on remote non-infarcted LV wall showed that myocyte cross-sectional area (MCSA), significantly increased in MI group compared to SHAM (MI vs SHAM: 357.8 ± 17.2 μm^2^ vs 187.3 ± 18.6 μm^2^; p < 0.001), whereas montelukast treatment preserved it (MI vs MI + MTK: 357.8 ± 17.2 μm^2^ vs 274.6 ± 26.2 μm^2^; p < 0.05) (Fig. 3A).

These results indicate that montelukast treatment preserves the ischemic LV tissue and exerts a cardio-protective effect on post-MI remodelling and healing.

### GSK3 protein expression

It’s known that PI3K/Akt pathway is activated in hypertrophic condition and it is generally assumed that signal transduction induced inhibition of GSK3 (through its phosphorylation). The inhibition of GSK3 protein activates transcription and translation factors promoting hypertrophic responses.

We observed in MI group a significant increase in pGSK3β/GSK3β compared to SHAM (MI vs SHAM: 3.45 ± 0.34 vs 1 ± 0.39; p < 0.01) and montelukast treatment was able to significantly counteract this increase (MI vs MI + MTK: 3.45 ± 0.34 vs 2.11 ± 0.92; p < 0.05) (Fig. 3B and Supplementary Information, Fig. 1S–5S). No statistically significant difference in GSK3β was observed between groups (SHAM: 1.0 ± 0.16; MI: 0.91 ± 0.07; MI + MTK: 1.01 ± 0.06, Anova p value = 0.48).

All these data suggest the development of hypertrophic condition in remote non-infarcted tissue after myocardial infarction and showed the ability of montelukast to preserve this maladaptive condition also sustaining contractility.

## Discussion

In this study, we demonstrated that the CysLT1 receptor antagonist, montelukast, reduces the severity of the long-term cardiac damage after MI, preventing the thinning of the infarct zone, preserving myocardium in the border zone, and limiting hypertrophy and fibrosis of the non-infarcted myocardium. The preserved cardiac functionality after an ischemic insult, even in the long term, gives hope for the repositioning of montelukast in the clinic.

Although it is known that the production of CysLTs is augmented following the development of MI^23^, there are still few studies concerning the cardioprotective effects of montelukast. *In-vitro*, montelukast reduced oxidative stress and apoptosis in LTC4-treated primary murine cardiomyocytes^19^. Instead, *in-vivo*, montelukast showed a beneficial effect on myocardium remodelling and myocardial function in a mouse model of cryo-induced left ventricular injury^19^and ameliorated doxorubicin-induced cardiotoxicity in rats, inhibiting ROS-mediated TNFα/NF-κB pathways and enhancing P-gp activity^24^. In addition, montelukast blunted the process of cardiac fibrosis and subsequently preserves cardiac function in a transverse aortic constriction (TAC) mouse model^22^. However, up to now, no study has investigated montelukast in a specific model of cardiac ischemia.

The main relevant finding of this study is the improvement elicited by montelukast in the cardiac remodelling and function at the late stage after MI. Using histological approaches and analysing the specific regional left ventricular function by cMRI, the changes occurring in individual areas of heart were studied in more detail.

First, we found that montelukast reduced the infarct size and improved the scar thickness ratio in the infarct zone.

The effect on the thinning of the ischemic zone is to be highlighted, since it increases the tensile strength of the infarcted wall, counteracting the expansion and adverse remodelling^25^. Relevantly, this morphological effect was accompanied by a functionally improvement of the LV anterior wall.

Several evidence indicate apoptosis and inflammation as key players in the myocardial loss after acute MI and in the process of subsequent left ventricular remodelling^26,27^.

Studies in MI mice clearly showed that cardiomyocyte apoptosis was correlated with wall thinning and inhibition of cardiomyocyte apoptosis lead to attenuation of wall thinning and improving ventricular remodelling^28,29^. Here, we demonstrated that montelukast suppresses the expression of the pro-apoptotic gene Bax and increase the expression of the anti-apoptotic gene Bcl-2. The current finding is in accordance with previous data obtained in animal models other than MI, in which montelukast was effective in inhibiting apoptosis^30–32^.

The results of the present study also indicated that montelukast protected from myocardial cell injury, reducing the production of pro-inflammatory cytokines in infarcted myocardium. In our hypothesis, this post-MI healing of the ischemic zone could be related to the anti-inflammatory and modulating macrophage polarization effects of montelukast. The anti-inflammatory effect of montelukast has recently been observed in several experimental models of ischemia/reperfusion (I/R) injury in kidneys, ovaries, brain, spinal cord and^33–37^. In the present study, we show, for the first time, that montelukast strongly reduced the mRNA expression of Il1β and Ccl2 in the ischemic area of mice subjected to permanent LAD ligation. The effect on Il1β could play a relevant role in the cardioprotection of montelukast. Indeed, the pharmacological blockade of IL-1 receptor reduced cardiomyocytes apoptosis and inhibited caspase-1 and caspase-9 activities in a mouse and rat model of myocardial infarction^38^. The genetic deletion of the type I IL-1 receptor reduced neutrophil and macrophages infiltration, mRNA expression of chemokine and cytokine, collagen deposition in the infarct and the remodelling peri-infarct area, and finally dilative remodelling in mice with cardiac ischemia/reperfusion (I/R) injury^39^. Similar results were reported for a specific anti-Il1β antibody which inhibited cardiomyocytes apoptosis, limited left ventricular enlargement and improved systolic dysfunction in a mouse model of non-reperfused MI^40^. Neutralizing Il1β also lowered the post-MI enhanced hematopoietic stem cell proliferation, which is responsible for the increased recruitment of monocytes, macrophages and neutrophils in the ischemic lesion, as shown in a parabiotic mouse model^41^. Monocytes and macrophages are key players in the inflammation and injury repair after MI^42^ and their excessive infiltration and activation is associated with augmented inflammation and myocardium injury, leading to infarct expansion and adverse LV remodelling in mice^43^. Thus, we hypothesize that, at least partially through its anti-inflammatory effect, and in particular the decreased mRNA expression of Il1β, montelukast can counteract the detrimental mechanisms occurring in the infarct and peri-infarct zones, such as the increased chemotaxis, vascular permeability, infiltration of immuno cells and release of inflammatory mediators, which lead to the exacerbation of the ischemic damage. Montelukast could also shift monocyte/macrophage polarization towards M2-like phenotype, thereby promote inflammation resolution, infarct healing and improved cardiac function. We have recently show that montelukast influenced the phenotype of macrophages, increasing the number of M2 polarized microglia/macrophages, over the M1 phenotype, at acute phase of permanent brain ischemia^14^. To corroborate this hypothesis, in CD226 KO mice, in which the polarization toward M2 phenotype is enhanced and the M1 polarization suppressed, the progressive ventricular dilation was attenuated and the cardiac function was ameliorated after MI^44^.

Second, montelukast also counteracted the development of wall thickening and fibrosis in the remote non-infarcted myocardium. In detail, we observed a reduced cardiomyocyte cross-sectional area and collagen content in MI mice treated with montelukast compared to vehicle. Relevantly, RFAC analysis highlighted that these histological effects were associated with an overall beneficial effect on ventricular remodelling and with improved heart function. Moreover, the early modulation of target genes involved in processes of cellular hypertrophy strengthen the hypothesis that montelukast protective effect on LV remote non-infarcted wall, occurring acutely after myocardial infarction, was partially liable for the functional effects of montelukast on the remote area at the late stage after MI.

In this study, we showed, for the first time, that montelukast can modulate the activity of GSK3β, a serine/threonine kinase which is involved in many cellular functions in the heart, including gene expression, hypertrophy and apoptosis^45,46^. GSK3β is typically active in un-stimulated cells and can be inactivated by phosphorylation. Relevantly, enhanced phosphorylation of GSK3β was found in fibrotic tissues from ischemic human and mouse hearts, and deletion of GSK3β from cardiac fibroblasts led to excessive fibrogenesis and scarring in ischemic hearts, impairing cardiac function^47^. In line, inducible cardiomyocyte-specific GSK3β KO mice with permanent MI displayed hypertrophy in the remote myocardium, but developed a better-preserved LV function and less dilatation^48^. Another study suggested that inhibition of GSK3β exacerbates ischemic injury but protects against I/R injury probably by modulating mTOR and autophagy^49^. This contrasting data suggests that GSK3β-mediated regulation of ventricular function in ischemic heart is complex, as overexpression/activation is detrimental but sustained systemic inhibition could be detrimental too. Thus, a compound able to modulate rather than inhibit the activation of GSK3β might be more appropriate in the setting of MI.

In summary, this study clearly demonstrated the ability of montelukast to prevent the development of deleterious cardiac remodelling after MI and consequently reducing functional impairment, by controlling excessive inflammation, preserving the thinning of ischemic wall and preventing cardiomyocyte hypertrophy in the non-ischemic wall. Our results support the repurposing of montelukast for the treatment of MI, even though we are aware that additional research is required to confirm our data and to gain a better understanding of the mechanisms involved.

## Methods

### Animals

This study was carried out in strict accordance with the ARRIVE and European Guidelines for Animal Care. The protocol was approved by the Committee on the Ethics of Animal Experiments of the University of Milan (approval number 479/2015-PR and 1097/2016-PR).

Animals were fed *ad* libitum with standard chow and water. All surgery was performed under anesthesia, and all efforts were made to minimize the number of animals used and their suffering.

**Figure 4:**
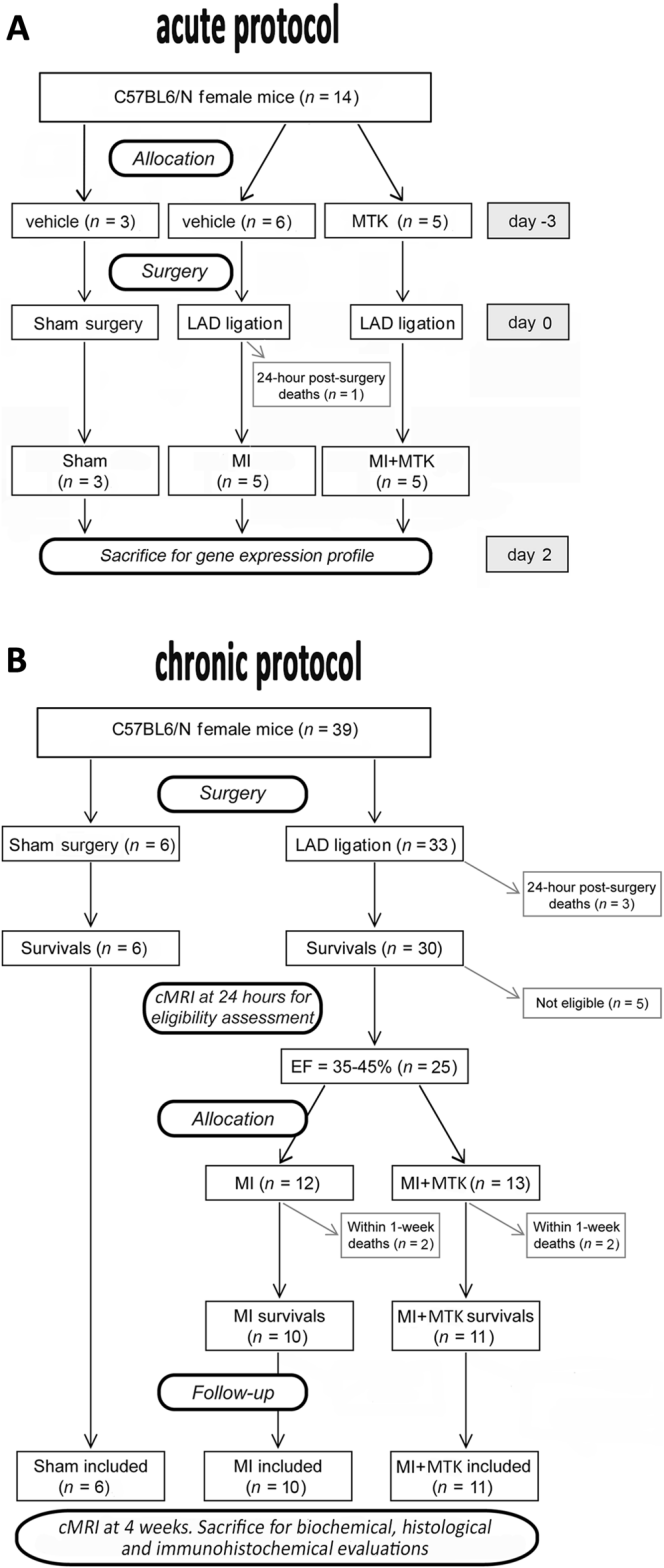
Flow chart showing the experimental protocols with the number of animals used, died and included in the study.

A total of 53 C57/BL6 female mice of 8–10 week old, weighting 18–20 g, purchased from Charles River (Calco, Italy), were used and enrolled in acute (n = 14) and chronic (n = 39) protocols (Fig. 4).

### Experimental protocols

All the experiments were conducted in the light phase. Mice were anesthetized by intraperitoneal injection of a mixture of ketamine (75 mg/kg) and medetomidine hydrochloride (1 mg/kg), endotracheally intubated in a supine position with a steel tube and ventilated with positive airway pressure (tidal volume of 140 ul at 150 breaths/min).

Mice were allocated to left anterior descending (LAD) coronary artery ligation or sham surgery, as previously described^50^. After surgery atipamezolo (2.5 mg/kg) was administered to encourage animal awakening, and then the animals were extubated, placed to warm cage and monitored until complete awakening.

### Acute protocol

Starting from 3 days before surgery mice were treated intraperitoneally with vehicle (MI, n = 5) or with montelukast sodium powder (Cayman Chemical, Ann Arbor, MI, United States), dissolved as previously described^51^ at the dose of 10 mg/kg/day (MI + MTK, n = 5) until sacrifice. Sham-operated mice were included as control (SHAM, n = 3). Forty-eight hours after surgery mice were sacrificed and the heart collected for biochemical analyses.

Of the 6 mice treated with vehicle and subjected to LAD ligation, 1 died before 24 h, whereas no death was observed in MI + MTK and SHAM groups (Fig. 4A).

### Chronic protocol

Twenty-four hours after surgery all survived mice (n = 36) were subjected to cardiac Magnetic Resonance Imaging (cMRI) and infarcted mice with an ejection fraction (EF%) within the range 35–45%^50^ were included into the study, and randomly assigned to two experimental groups: *i*) treated intraperitoneally with vehicle (MI, n = 10); ii) treated intraperitoneally with montelukast sodium powder (Cayman Chemical, Ann Arbor, MI, United States), at the dose of 10 mg/kg/day (MI + MTK, n = 11) for 4 weeks. All sham-operated mice were included as control (SHAM, n = 6).

Four weeks after surgery all the animals were subjected to cMRI, then the mice were sacrificed, and the heart collected for biochemical, histological and immunohistochemical evaluations.

Of the 33 mice that underwent LAD ligation 3 died before 24 h, 5 were excluded because their EF was out of 35–45% range at 24 h and 4 died within 1 week after randomization (2 MI and 2 MI + MTK). No other death was observed in the remaining follow-up period and in SHAM group (Fig. 4B).

### Cardiac magnetic resonance imaging

Cardiac magnetic resonance imaging was performed using a 4.7 T vertical-bore MR magnet (Bruker spectrometer AMX3 with micro-imaging accessory, Germany). Anesthesia was induced by exposing mice to 3% isoflurane (Merial, Toulouse, France) in 100% oxygen in an induction chamber. Mice were then fixed on a holder, anaesthetized with inhaled 1% isoflurane, and placed into the vertical, 15-cm bore spectrometer equipped with a 3.8 cm diameter birdcage coil. During the acquisitions, the respiration was monitored.

A series of scout images were first acquired to define landmark points for the acquisition of the long-axis 4 chamber view and then, orthogonally to it, the short-axis sections^52^.

The images were acquired using a retrospective gated cine gradient echo sequence with the following parameters: and a retrospective gated cine gradient echo sequence with the following parameters: echo time (TE) 1.9 ms; repetition time (TR) 10 ms; field of view 4 × 4 cm^2^; acquisition matrix 128 × 128 pixels; slice thickness 1.3 mm; 6–8 axial slices spaced 1 mm to fully cover the left ventricular (LV).

Then, the images were analyzed using custom software implemented in Python, along the lines of Franzosi et al.^52^ in order to obtain LV parameters as end-diastolic (EDV), end-systolic (ESV) and stroke volumes (SV), ejection fraction (EF).

Mid papillary images were analyzed in order to measure wall thicknesses (WT) of infarcted (anterior), border zone (border) and posterior (remote) wall and derived ventricular mass.

In detail, on mid papillary frames, epicardial and endocardial borders were manually traced and regional LV thickness was obtained by tracing 18 concentric rays (one every 20°, starting from the ray connecting LV centroid to a point placed at the junction between the right ventricular free wall and the interventricular septum). Diastolic and systolic wall thickness as mean distance between the endocardial and epicardial borders along the three consecutive rays for each zone (infarcted, border and posterior) were computed^52^.

For the quantitative analysis of regional function, the LV cavity was divided into six 60° sectors (anterior, antero-septal, septal, lateral, posterior and inferior) and regional fractional area change % (RFAC) was then computed for each segment as [(end diastolic area—end systolic area)/end diastolic area × 100] and used as index of regional contractility. Regional LV wall motion was displayed in a "bull’s eye" format, and to average the RFAC in mice with a different number of slices covering the LV, RFAC data were resampled using cubic spline interpolation to 10 slices for each of the six sectors.

### Tissue collection

To prepare specimens for histological analysis, abdominal aorta was cannulated and heart was arrested in diastole, with 2 mL of a solution of 0.1 M CdCl2 and 1 M KCl^53^, and retrogradely perfused with 0.01 M phosphate saline buffer (PBS) and then with 4% (vol/vol) phosphate-buffered formalin for 10 min each. Hearts were collected and postfixed in 4% phosphate-buffered formalin for 24 h and embedded in paraffin. Consecutive 8 $\upmu$m heart axial sections (from base to apex) were prepared.

For protein and gene expression analyses, hearts were retrogradely perfused for 10 min with 0.01 M PBS and left ventricle infarcted and non-infarcted wall were collected separately and stored at -80 °C. For gene expression analyses, RNA were stabilized and protected maintaining tissue in RNAlater (Life Technologies, Carlsbad, CA) for 14 days at 4 °C before freeze.

### Determination of infarct size, morphometry and cardiomyocytes’ cross-sectional area

For collagen staining, deparaffinized and rehydrated heart sections were incubated in 0.1% Sirius Red Solution (Direct Red 80, Sigma-Aldrich, St. Louis, MO) in picric acid for 45 min, then washed, dehydrated (1 min each in 70%, 96%, and absolute ethylic alcohol, and then 10 min in xylene), and mounted with DPX mountant for microscopy. Images were acquired with a high-resolution digital camera using 1:1 macro-lens.

Infarct size was determined on Sirius red stained cardiac axial section (one for each mm) along the apex-basis axis. To define the infarct lengths, LV endocardial infarct length was taken as the length of LV endocardial infarct scar surface that included greater than 50% of the whole thickness of myocardium, and LV epicardial infarct length as the length of the transmural infarct region. Epicardial infarct ratio was obtained by dividing the sum of epicardial infarct lengths from all sections by the sum of LV epicardial circumferences from all sections. Endocardial infarct ratio was calculated similarly. Infarct size derived from this approach was calculated as [(epicardial infarct ratio + endocardial infarct ratio)/2] × 100^54^.

The scar thickness (mean of five equidistant measurements) and septum thickness (mean of three equidistant measurements) were measured in a middle section of the heart^55^.

Myocytes’ cross-sectional area (MCSA) was determined on 3 cardiac sections from each heart stained with germ agglutinin–Alexa Fluor 488 (50 mg/mL in 0.01 M PBS, Invitrogen, Waltham, MA, USA) overnight at 4 °C, then incubated with Hoechst 33258 nuclear stain (Invitrogen, Waltham, MA, USA), and then mounted using fluorescence mounting medium (Dako, Milan, Italy). Images from the non-infarcted region (432 μm × 324 μm) were acquired using a fluorescence microscope (Axiovert 200, Zeiss, Jena, Germany). Cross-sectional area of 100–150 cardiomyocytes from each mouse was measured on transversally sectioned cells with circularity greater than 0.6 and round nuclei^56^.

All quantitative analyses were performed in blind using Photoshop CS6 (Adobe System) or Image J (U.S. National Institutes of Health, Bethesda, MD, USA).

### Real-time PCR

Total RNA from cardiac tissue was extracted with TRIzol Reagent (Sigma-Aldrich, St. Louis, MO) according to manufacture’s instructions. RNA samples were quantified with a GE Healthcare NanoVue Plus UV–Vis Spectrophotometer and 1 μg of RNA was reversely transcribed into cDNA by using SensiFAST™ cDNA Synthesis Kit (Meridian Bioscience Cincinnati, Ohio). A quantitative PCR was carried out by SsoAdvancedTM Universal SYBR Green Supermix (Bio-Rad) using Bio-Rad CFX Connect real-time System (Bio-Rad). Each experiment was run in triplicate and changes in mRNA levels were expressed as 2^−ΔΔCt^ values and presented relative to the mean of the RPL13a reference gene. Sequences of the PCR primers are shown in Table 2, and PCR conditions were set as follows: initial denaturation at 95 °C for 3 min, followed by 39 cycles at 95 °C for 10 s and 60 °C for 30 s.

**Table 2:** Primer sequences of the analyzed genes.

Gene		
Rpl13a	Fwd	ACAGCCACTCTGGAGGAGAA
	Rev	GAGTCCGTTGGTCTTGAGGA
Il1β	Fwd	CGTTCCCATTAGACAACTGC
	Rev	GCTCATGGAGAATATCACTTGT
TNFα	Fwd	GCCTCTTCTCATTCCTGCTT
	Rev	AGGGTCTGGGCCATAGAACT
Ccl2	Fwd	TGCCCTAAGGTCTTCAGCAC
	Rev	AAGGCATCACAGTCCGAGTC
Bcl-2	Fwd	GACGCGAAGTGCTATTGGT
	Rev	TCAGGCTGGAAGGAGAAGAT
BAX	Fwd	CTACAGGGTTTCATCCAG
	Rev	CCAGTTCATCTCCAATTCG
NPPA	Fwd	CAAAGGCTGAGAGAGAAACC
	Rev	GCCAGGAAGAGGAAGAAGC
NPPB	Fwd	GCCAGTCTCCAGAACAATCC
	Rev	AGGCGCTGTCTTGAGACCTA
MYH7	Fwd	GCCTACCTCATGGGACTGAA
	Rev	ACATTCTGCCCTTTGGTGAC

### Western blot analysis

Myocardial LV samples were pulverized in liquid nitrogen, and then a portion (60–90 μg) was homogenized by dispenser (T 25 ULTRA-TURRAX – IKA, Germany) on ice in 10 volumes of RIPA lysis buffer (150 mM NaCl, 10 mM, Tris, pH 7.4, 5 mM EDTA, 0.1% SDS, 1% sodium deoxycholate and 1% Triton X-100) added with protease inhibitors (Roche Diagnostics, Germany) and phosphatase inhibitors (Sigma-Aldrich, Germany) according to manufacture’s instructions. The homogenates were spin (13.000 rpm for 15 min at 4 °C) and the supernatants were collected and assayed for protein quantification by Bradford method^57^. Aliquots of the protein extracts were prepared into single-use samples and stored at − 80 °C until use. An aliquot of 50 μg of protein from each sample was separated on 10% SDS–polyacrylamide gel by electrophoresis and then transferred to nitrocellulose membranes using iBlot 2 Dry Blotting System (Thermo Fisher Scientific, USA). The membranes were reversibly stained with Ponseau S and then blocked with 5% non-fat dry milk in TBS-TWEEN 20 0.1% at room temperature for 1 h. Subsequently, the membranes were incubated with primary antibodies (all prepared in 5% non-fat dry milk in TBS-TWEEN 20 0.1%) rabbit anti GSK3-α/β (1:1000, Cell Signaling, MA, USA, cat n°5676), mouse anti p-GSK3-β (1:500, Cell Signaling, MA, USA, cat n°14630) and rabbit anti GAPDH (1:500, Santa Cruz, CA, USA, cat n° sc-25778) overnight at 4 °C with gentle shaking. Next, the membranes were washed and incubated with goat anti mouse (Bio-Rad Laboratories, USA) or anti rabbit (Sigma Aldrich, Germany) HRP-labeled secondary antibody (1:2500 in 5% non-fat dry milk in TBS-TWEEN 20 0.1%) at room temperature for 2 h. The blots were visualized using ECL solution (Cyanagen, Italy). Finally, the reaction products were densitometrically quantified using an Odyssey imaging system (LI-COR Biosciences, USA) and Image J software. The ratio of the protein of interest was subjected to GAPDH, which acted as the internal control in the experiments.

### Statistical analysis

For each experimental procedure, the proper sample size has been calculated by means of G*Power 3.1 software, estimating effect size and standard deviation based on previously published data^50^ and fixing the alpha value (type 1 error) at the level of 5% (p = 0.05) and the power at 80%.

Data are expressed as mean ± standard error of the mean (SEM). Statistical analysis was performed using Prism 7 software (GraphPad, San Diego, CA, USA). For multiple comparison, One-way ANOVA parametric tests followed by a Dunnett “post hoc” analysis was performed. For infarct size and MRI data of left ventricle 24 h after surgery, unpaired t Test was performed. Differences were considered significant for p value < 0.05.

### Supplementary Information

Supplementary Information.

## Data Availability

All data that support the findings of the current study can be given upon reasonable request to corresponding author (Prof. Luigi Sironi).
