# Supplementary material for: Montelukast, an available and safe anti-asthmatic drug, prevents maladaptive remodelling and maintains cardiac functionality following myocardial infarction
Source: Sci Rep. 2024 Feb 9;14:3371. doi: 10.1038/s41598-024-53936-x (PMC10858037; doi:10.1038/s41598-024-53936-x)
Supplement: Supplementary file 1 — Supplementary Information. [file 41598_2024_53936_MOESM1_ESM.docx]

**Supplementary Information**

The original images of full-length blots cannot be provided because the blots were cut prior to hybridisation with antibodies.

Images of all blots for all replicates performed for pGSK3β, GSK3β and GAPDH are reported.

A (pGSK3β, MW 46 KDa)

1 2 3 4 5 6 7 8 9 10 11 12 13 14


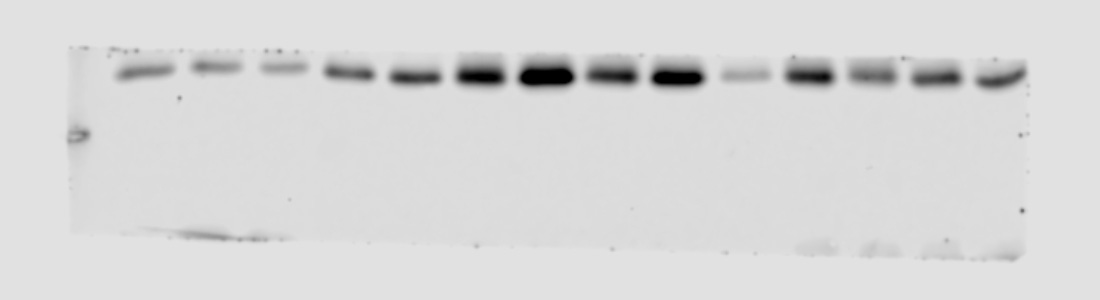


B (GSK3β, MW 46 KDa)


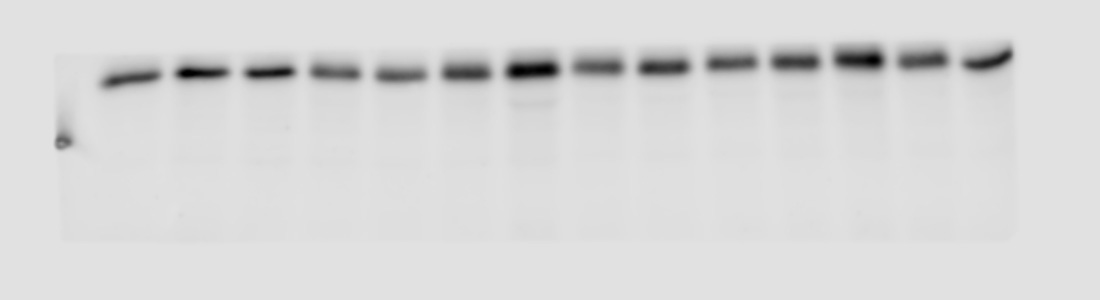


C (GAPDH, MW 37 KDa)


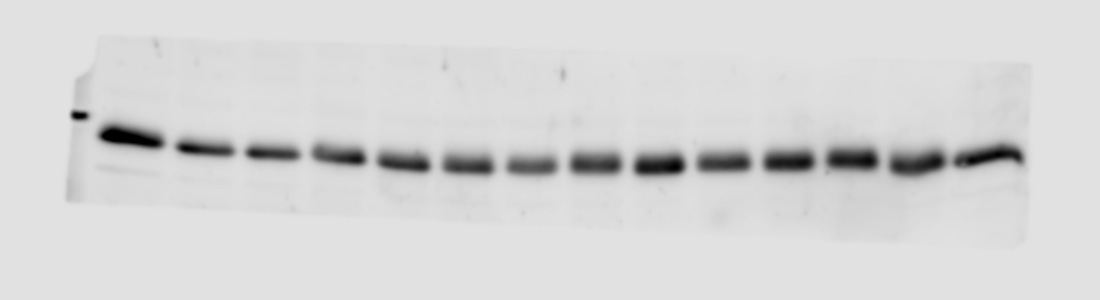
**Figure 1S**

Western blot membranes for pGSK3β (A), GSK3β (B) and GAPDH (C).

Marker is 37 KDa, lines 1-3 Sham group (black bracket), lines 4-9 MI group (green bracket), lines 10-14 MI + MTK group (red bracket).

The regions of the original blots used in main figure were denoted using red boxes.

A (pGSK3β, MW 46 KDa)


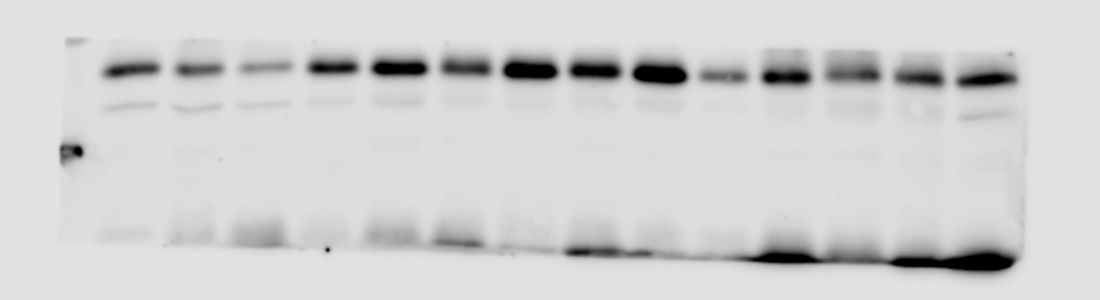


1 2 3 4 5 6 7 8 9 10 11 12 13 14

B (GSK3β, MW 46 KDa)


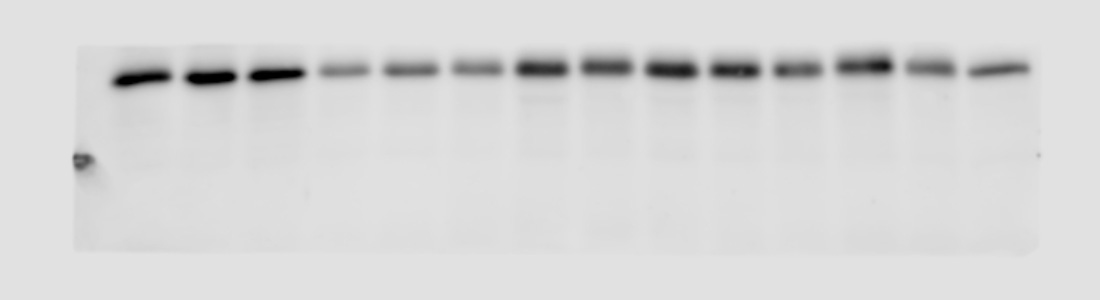


C (GAPDH, MW 37KDa)


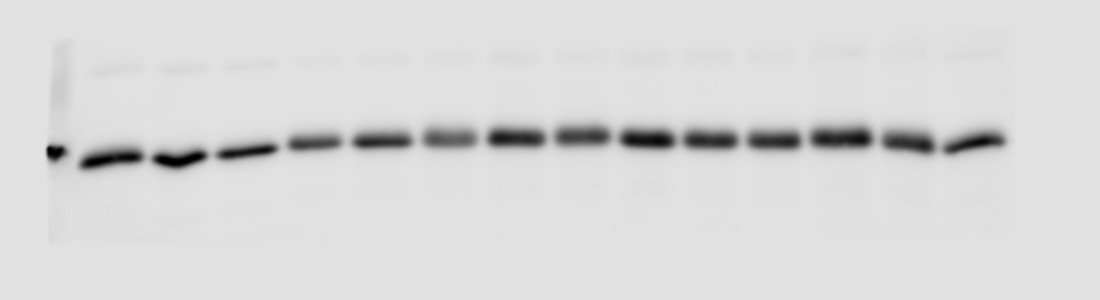


**Figure 2S**

Western blot membranes for pGSK3β (A), GSK3β (B) and GAPDH (C).

Marker is 37 KDa, lines 1-3 Sham group (black bracket), lines 4-9 MI group (green bracket), lines 10-14 MI + MTK group (red bracket).

A (pGSK3β, MW 46 KDa)

1 2 3 4 5 6 7 8 9 10 11 12 13 14


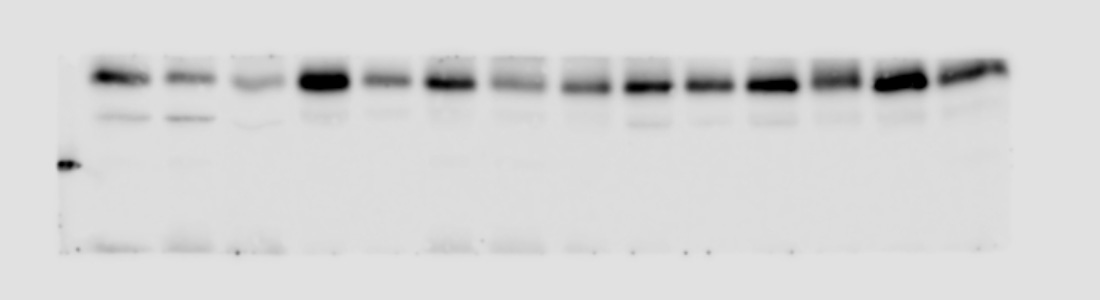


B (GSK3β, MW 46 KDa)


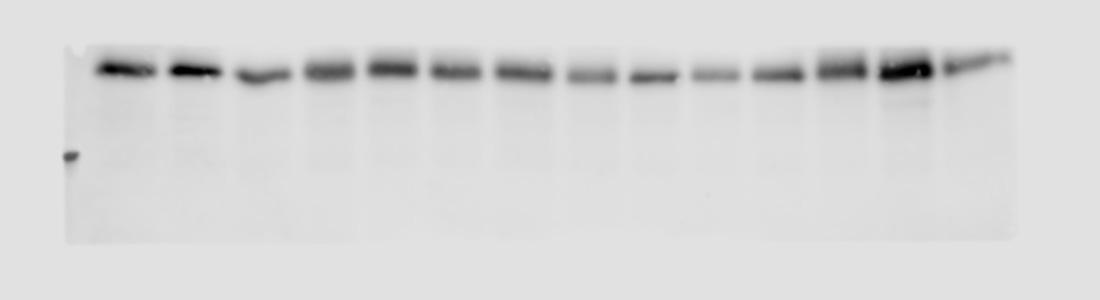


C (GAPDH, MW 37 KDa)


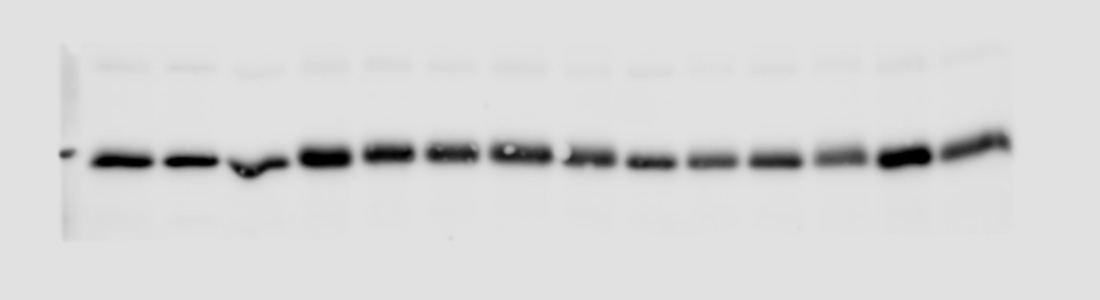


**Figure 3S**

Western blot membranes for pGSK3β (A), GSK3β (B) and GAPDH (C).

Marker is 37 KDa, lines 1-3 Sham group (black bracket), lines 4-9 MI + MTK group (red bracket), lines 10-14 MI group (green bracket).

A (pGSK3β, MW 46 KDa)

1 2 3 4 5 6 7 8 9 10 11 12 13 14


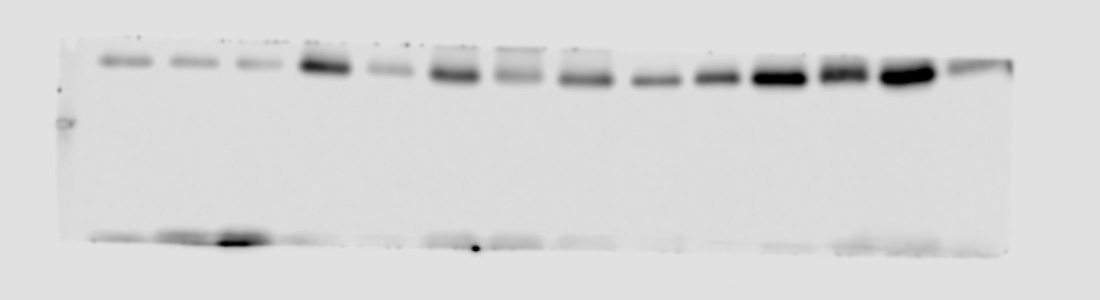


B (GSK3β, MW 46 KDa)


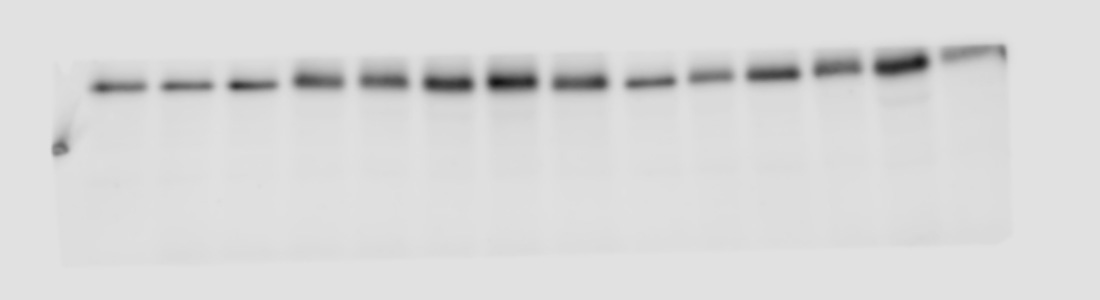


C (GAPDH, MW 37 KDa)


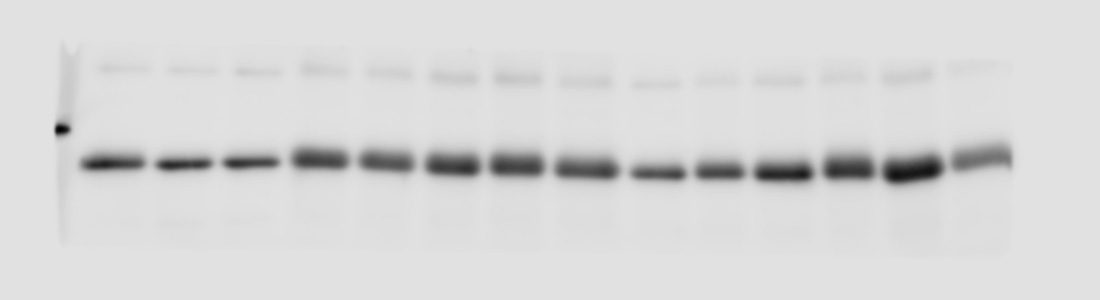


**Figure 4S**

Western blot membranes for pGSK3β (A), GSK3β (B) and GAPDH (C).

Marker is 37 KDa, lines 1-3 Sham group (black bracket), lines 4-9 MI + MTK group (red bracket), lines 10-14 MI group (green bracket).

A (pGSK3β, MW 46 KDa)

1 2 3 4 5 6 7 8 9 10 11 12 13 14


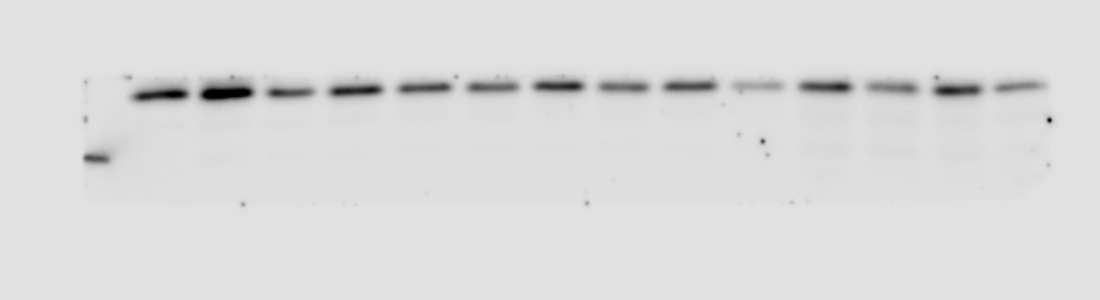


B (GSK3β, MW 46 KDa)


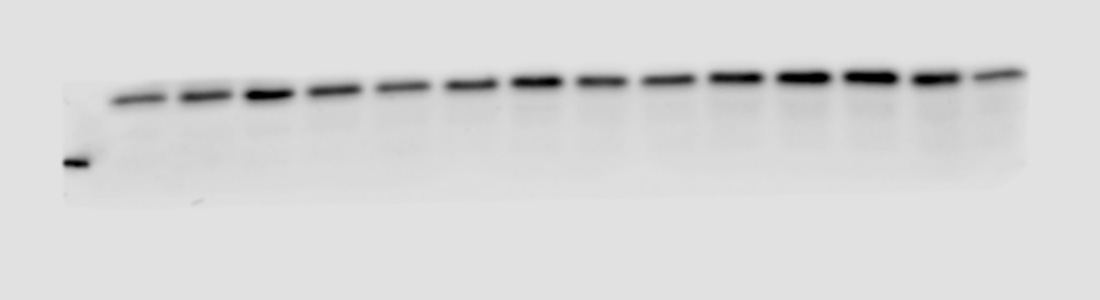


C (GAPDH, MW 37 KDa)


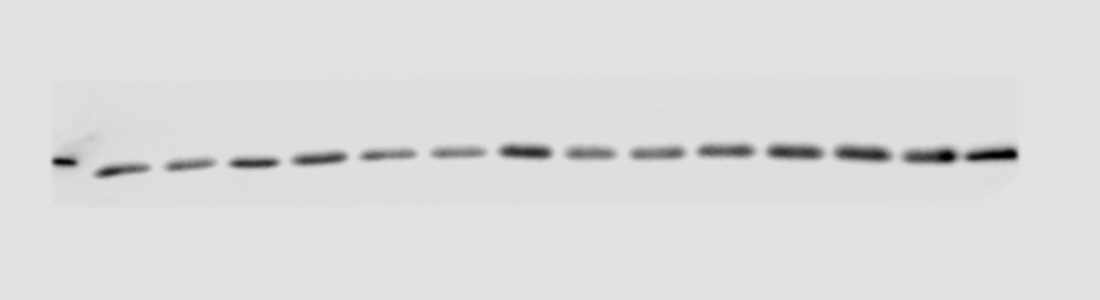


**Figure 5S**

Western blot membranes for pGSK3β (A), GSK3β (B) and GAPDH (C).

Marker is 37 KDa, lines 1-5 MI group (green bracket), lines 6-8 Sham group (black bracket), lines 9-14 MI + MTK group (red bracket)
